# Viral Threats to Australian Fish and Prawns: Economic Impacts and Biosecurity Solutions—A Systematic Review

**DOI:** 10.3390/v17050692

**Published:** 2025-05-10

**Authors:** Md. Mizanur Rahaman, Bhavya Sharma, Saranika Talukder, Muhammad Jasim Uddin, Muhammad A. B. Siddik, Subir Sarker

**Affiliations:** 1 Biomedical Sciences and Molecular Biology, College of Medicine and Dentistry, James Cook University, Townsville, QLD 4811, Australia; mdmizanur.rahaman@my.jcu.edu.au; 2 School of Health & Biomedical Sciences, STEM College, RMIT University, Bundoora, VIC 3083, Australia; bhavyasharma731@gmail.com; 3 College of Science and Engineering, James Cook University, Townsville, QLD 4811, Australia; saranika.talukder@jcu.edu.au; 4 School of Veterinary Medicine, Murdoch University, Perth, WA 6150, Australia; jasim.uddin@murdoch.edu.au; 5 Centre for Biosecurity and One Health, Harry Butler Institute, Murdoch University, Perth, WA 6150, Australia; 6 Nutrition and Seafood Laboratory (NuSea.Lab), School of Life and Environmental Sciences, Deakin University, Queenscliff, VIC 3225, Australia; m.siddik@deakin.edu.au

**Keywords:** viruses, fish, prawn, economic impact, biosecurity

## Abstract

Viral diseases pose significant threats to aquaculture industries worldwide, including the Australian fish and prawn farming sectors, which contribute over AUD 1.6 billion annually to the national economy. The Australian aquaculture industry relies heavily on wild-caught broodstock for seedstock production, introducing substantial and unprecedented biosecurity risks. This systematic review consolidates current knowledge on the viral pathogens affecting key Australian fish and prawn species, their economic impacts, and the biosecurity measures implemented for mitigation. Notably, viral outbreaks have led to losses exceeding AUD 100 million in some sectors, highlighting the urgent need for improved management. Existing biosecurity strategies, including surveillance systems, molecular diagnostics, and pathogen exclusion protocols, are critically assessed for their effectiveness. Emerging approaches such as genetic resistance breeding, advanced vaccination technologies, and integrated risk management frameworks are also explored. Key knowledge gaps, particularly in the context of emerging viral pathogens and their ecological interactions under changing environmental conditions, are identified as priority areas for future research. This review emphasises the necessity of adopting a multidisciplinary approach to enhance the resilience of Australian aquaculture, advocating for stronger biosecurity frameworks and innovative technologies to mitigate the escalating risks posed by viral diseases.

## 1. Introduction

Australia is renowned for its diverse marine species, hosting approximately 33,000 species within its marine ecosystems [1]. Over the past 50 years, the production of aquatic animals has increased from 33.65 million tons to 214.92 million tons and is projected to increase further [2,3]. Aquaculture originates from ancient Chinese wisdom and has progressively advanced into one of the most efficient methods of food production [4]. It supplies nearly one-third of the animal protein consumed in China and contributes approximately 60% of global aquaculture production [5].

While these figures illustrate the global importance of aquaculture, Australia represents a unique and critical case due to its vast marine biodiversity, geographic isolation, and reliance on both wild-sourced and farmed aquatic species [6]. Understanding the local context is essential to addressing the challenges unique to Australian aquaculture systems.

Fish and prawns are recognised as critical and sustainable nutritional resources, particularly in the contexts of the growing global population, the impacts of climate change, and health concerns, as they provide essential protein and nutrients, which is especially important in regions where alternative food sources are limited [7]. However, the increasing demand for these resources has driven the rapid growth of aquaculture, which has, in turn, facilitated the spread and emergence of highly pathogenic viruses that are frequently transmitted globally through aquaculture practices [8].

While the demand for seafood continues to rise, the expansion of the aquaculture industry faces significant constraints, limiting its capacity to fully meet market demands. Various aquatic species are cultivated at high densities in diverse water aquaculture systems, such as ponds, tanks, and cages. Intensive farming exposes aquatic species to stressful conditions, increasing their vulnerability to diseases and mortality [9]. Despite technological advancements in aquaculture production, achieving profitability remains a big challenge. Aquaculture technologies require higher investments in maintaining water quality and have a strong focus on preventing viral diseases [7]. Improper farming practices, combined with the challenges inherent to aquaculture, contribute to the rapid spread of diseases and diminish the ability of aquatic species to resist infections [9].

Both long-standing and newly emerging viral pathogens pose significant challenges, as the development of effective treatments remains elusive [9]. Additionally, co-infections involving multiple viral pathogens are emerging as a significant concern in aquaculture systems. These co-infections—such as white spot syndrome virus (WSSV) with infectious hypodermal and haematopoietic necrosis virus (IHHNV) in prawns [10] or salmonid alphavirus (SAV) alongside piscine orthoreovirus (PRV) in fish—can exacerbate disease severity, complicate diagnostics, and hinder effective disease management [11]. Despite their critical impact, co-infections remain under-investigated in the context of Australian aquaculture, representing an important gap in current surveillance and biosecurity frameworks.

Nevertheless, significant progress has been made in areas such as genetic breeding, seed industry advancement, nutrition and feed development, disease diagnosis and prevention, environmental protection, ecological engineering, and modernisation of aquaculture infrastructure, all of which contribute to enhanced disease control and the overall sustainability of the industry [4,12,13].

Diagnostic methods such as real-time PCR (qPCR) are commonly employed to detect the specific pathogens associated with disease outbreaks [14]. qPCR provides high sensitivity and specificity, enabling the quantification and typing of pathogens that impact Australia’s marine environment [15]. Emerging epizootics frequently cause significant losses in fish and prawn populations, leading to substantial economic impacts in commercial markets and posing serious threats to valuable aquatic animal stocks [16,17]. The severity of viral diseases is frequently exacerbated by delayed diagnoses, insufficient identification of causative agents, and inadequate understanding of critical epidemiological factors [18]. Although Australia is renowned for its pristine environment, strong biosecurity, and high terrestrial and aquatic animal production yields, implementing appropriate biosecurity in each industry remains a challenge. Thus, insufficient biosecurity is often a contributory factor to disease outbreaks and mass mortality events in aquatic populations [19].

Moreover, the Australian aquaculture industry heavily relies on wild-sourced broodstock for the black tiger prawn (*Penaeus monodon*), which presents considerable biosecurity risks. A recent study analysed 7472 pleopod samples from broodstock using TaqMan qPCR to detect viral pathogens. The study revealed a high prevalence of IHHNV (30%) and gill-associated virus (GAV) (28.1%) [20]. Reliance on wild-sourced broodstock for *P. monodon* farming exposes the industry to biosecurity risks, which can affect economic outcomes due to challenges related to domestication and genetic selection.

Despite ongoing advancements in disease diagnosis, genetics, and aquaculture infrastructure, significant knowledge gaps remain. In particular, the full economic and ecological implications of emerging and re-emerging viral threats in Australian aquaculture are not yet well understood. Moreover, the role of viral co-infections and their compounding effects on disease outcomes remain underexplored, complicating disease management and policy development. This review addresses existing knowledge gaps by systematically updating the current understanding of the viral diseases affecting Australian fish and prawns, with a focus on the economic impacts, co-infection dynamics, and biosecurity challenges. It also critically evaluates existing biosecurity policies and proposes innovative, evidence-based strategies for disease prevention, early detection, and sustainable aquaculture management. By doing so, this review aims to enhance the resilience of Australia’s aquaculture sector against present and future threats.

## 2. Materials and Methods

This systematic review investigates the viral pathogens affecting fish and prawns in Australia, assessing their economic impacts and associated biosecurity measures. The review follows the PRISMA 2020 guidelines to ensure a standardised and transparent reporting process (Supplementary Table S1) [21].

### 2.1. Information Sources and Search Strategy

A comprehensive literature search was conducted across five major electronic databases: Google Scholar, MEDLINE, PubMed, Web of Science, and Scopus. The search included studies published up to January 2025. The keywords used were virus, fish, prawn, pathogen, biosecurity, and economic impacts of viral diseases in fish and prawn. Only articles published in English and focused on Australian aquaculture were included. Duplicate records were identified and removed using EndNote software, followed by manual verification.

### 2.2. Inclusion and Exclusion Criteria

#### 2.2.1. Inclusion Criteria

The following inclusion criteria were used in this systematic review:

1. Investigated viral pathogens affecting fish and prawns in Australia.
2. Focused on biosecurity practices and/or the economic impacts of viral diseases in Australian aquaculture.
3. Included research conducted through in vivo, in vitro, or epidemiological studies, whether or not the mechanism of action was elucidated.

#### 2.2.2. Exclusion Criteria

The exclusion criteria listed below were carefully implemented to ensure the thoroughness and applicability of this systematic review:

1. Were duplicated or did not meet inclusion criteria upon title/abstract screening.
2. Focused on non-viral pathogens or unrelated aquaculture animals.
3. Addressed topics outside the scope of biosecurity and economic impacts in terms of Australian fish and prawn farming.

### 2.3. Risk of Bias in Individual Studies

**Figure 1:**
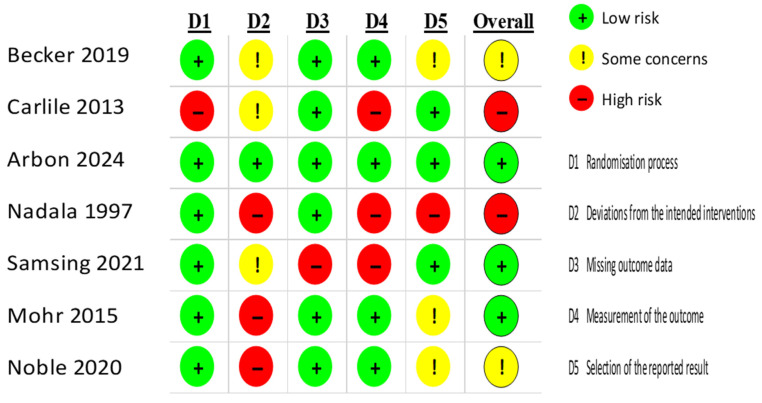
Risk of bias assessment. According to the Cochrane risk-of-bias tool for randomised trials (RoB 2): +, low risk of bias; −, high risk of bias; !, some risk of bias [22].

To assess the reliability of the results from the selected studies, we used the Cochrane risk-of-bias assessment criteria, specifically the RoB 2 tool version, dated 22 August 2019. This tool evaluates the risk across several domains: randomisation process, deviations from intended interventions, missing outcome data, outcome measurement, selection of reported results, and other potential sources of bias. Each category comprises questions across these domains, with the responses categorised as “YES” (indicating a low risk of bias, colour-coded green), “NO” (indicating a high risk of bias, colour-coded red), or “Some Concern” (indicating an uncertain risk of bias, colour-coded yellow) (Figure 1).

### 2.4. Study Selection Process

**Figure 2:**
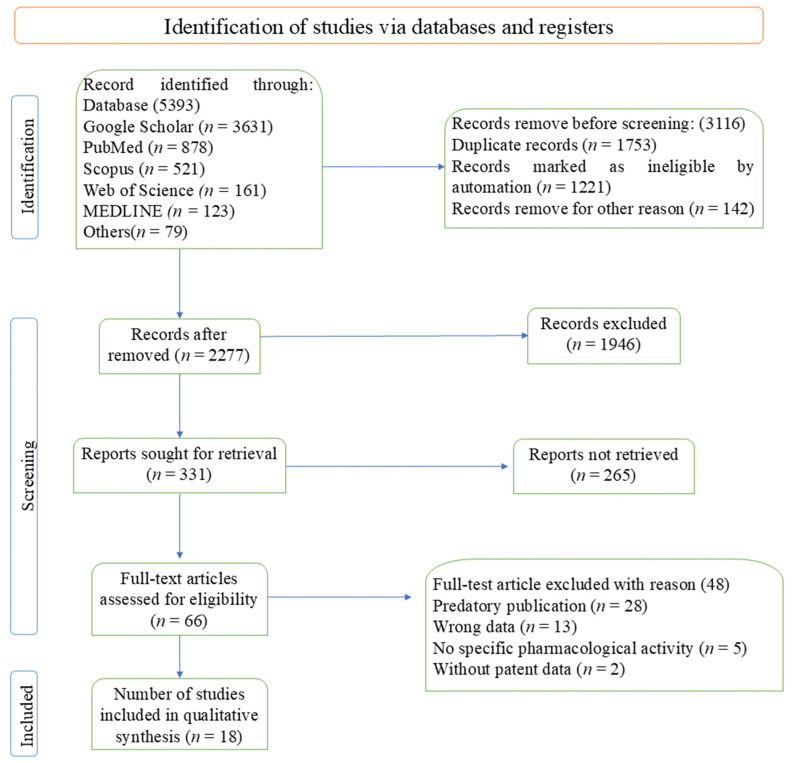
PRISMA flow diagram based on the data extraction.

After the initial database searches, 5393 records were identified. Following duplicate removal, 2277 records were screened based on the titles and abstracts. Subsequently, 331 full-text articles were assessed for eligibility. After retrieved 265 removed and selected 66 for further study. Finally excluding 48 articles on the basis of qualitative synthesis and 18 studies were included in the final manuscript. The full study selection process is illustrated in a PRISMA flow diagram (Figure 2).

### 2.5. Data Extraction and Characteristics of Sources

Data were extracted using a standardised form, focusing on the virus type, host species, disease mechanisms, environmental interactions, economic impacts, and biosecurity measures. Special attention was paid to emerging viral pathogens and mitigation strategies under changing environmental conditions.

## 3. Results and Discussion

### 3.1. Pathogenic Viruses in Fish

Eight fish viruses were identified via the search process. Table 1 lists the nomenclature, source, and year of publication for seven of these viruses. One virus has been excluded from Table 1 due to insufficient data. However, a description has been added for clarification.

#### 3.1.1. Epizootic Haematopoietic Necrosis Virus (EHNV)

Epizootic haematopoietic necrosis virus (EHNV), a member of the family *Iridoviridae* and genus *Ranavirus*, is a significant pathogen that causes severe necrosis of haematopoietic tissues in fish [17]. EHNV is a large icosahedral virus, approximately 175 nm in size, with a double-stranded DNA genome of approximately 127 kb [9]. In 1984, EHNV was first detected in Victoria. It has caused epidemic mortality in *P. fluviatis* and mild disease among farmed rainbow trout (*Oncorhynchus mykiss*) over the last two decades across New South Wales (NSW), the Australian Capital Territory (ACT), and South Australia [23]. EHNV primarily infects *P. fluviatilis*, though it is also capable of infecting a broad range of native and non-native fish species under experimental conditions [31]. Variable levels of infection and mortality were observed in mosquito fish (*Gambusia affinis*), Australian bass (*Macquaria novemaculeata*), Atlantic salmon (*S. salar*), and other perch species, including Macquarie perch (*Macquaria australis*), golden perch (*Macquaria ambigua*), and silver perch (*Bidyanus bidyanus*) under controlled laboratory conditions [32]. Infected fish displayed clinical symptoms including an enlarged abdomen, darkened skin, petechiae, and haemorrhages at the gill and fin bases [33]. Infection with EHNV also led to multifocal necrosis of haematopoietic tissue in the liver, spleen, and kidney of infected fish [34]. EHNV is known to be transmitted between susceptible hosts within a population via transfer through water or ingestion of tissues from infected fish. In the aquaculture industry, the primary mode of transmission was likely the movement of infected trout fingerlings, which may carry subclinical infections in a small proportion of individuals [35]. However, a recent report, including cases from 2021 at Lake Hume Resort, indicated that EHNV continues to be a concern, particularly with environmental changes facilitating the movement of carrier species like *P. fluviatilis* into new habitats [36]. The diagnostic methods for EHNV have evolved to include antibody detection via Western blot analyses, antigen-capture enzyme-linked immunosorbent assays (ELISAs), and polymerase chain reaction (PCR) techniques [37]. Controlling infection and preventing spread through active surveillance of fish health using biosecurity guidelines are recommended to manage EHNV, as there is no commercial vaccine available [38].

#### 3.1.2. Cyprinid Herpesvirus (CyHV)

The cyprinid herpesvirus is classified under the species *Cyprinid herpesvirus 1* (CyHV-1), within the genus *Cyprinivirus* of the *Alloherpesviridae* family, which belongs to the order *Herpesvirales* [39]. Two other major cyprinid species viruses have been identified, *Cyprinid herpesvirus 2* (CyHV-2, also called goldfish haematopoietic necrosis virus) and *Cyprinid herpesvirus 3* (CyHV-3, also referred to as koi herpesvirus (KSV)) [40,41]. In 2003, CyHV-2 was first detected in Western Australia; it originated from goldfish (*Carassius auratus*) that had been cleared from quarantine and subsequently transmitted the virus to domestic fish through the live fish trade [24]. Previous studies have highlighted that CyHV-2 exemplifies how aquatic pathogens from the ornamental fish trade could infiltrate and spread within farmed and wild populations, emphasising its critical significance for risk assessments of megalocytivirus infection in live ornamental fish imported into Australia [24,42]. CyHV-3 is distinguishable from CyHV-1, the causative agent of carp pox, and CyHV-2 based on differences in the clinical signs, host range, antigenic properties, growth characteristics, cytopathic effects (CPEs) in cell culture, and DNA sequencing [43]. CyHV-3 has been detected in goldfish (*C. auratus*) and crucian carp (*Carassius carassius*) [44,45,46]. Infected fish typically exhibit severe gill necrosis, pale gills and skin, and skin haemorrhages, with the disease progression varying according to the environmental conditions, accelerating in summer and slowing in winter [47]. PCR and ELISA have been employed to detect CyHV viral DNA and viral antigen (Ag) in faeces [48]. To control the endemic spread of CyHV2 in domestic farms, the Australian Government Department of Agriculture, Fisheries and Forestry revoked the requirement for goldfish exported to Australia to be certified free of CyHV2 [24].

#### 3.1.3. Tasmanian Atlantic Salmon Reovirus (TSRV)

Tasmanian Atlantic salmon reovirus (TSRV) is a member of the genus *Aquareovirus*, belonging to the family *Reoviridae*. TSRV possesses an 11-segment dsRNA genome, similar to those of viruses in the genera *Mycoreovirus* and *Rotavirus* [49]. In 1990, TSRV was first isolated in Tasmania from *S. salar* [25]. A previous study reported that TSRV is non-pathogenic; however, it still has an impact on fish production [50]. According to Carlile [25], there was increased mortality in *S. salar* infected with TSRV, especially when the temperature increased above 18 °C; thus, the incidence was higher during summer. The temperature conditions of Tasmanian waters have historically been favourable for the growth of Atlantic salmon; however, as water temperatures have risen due to climate change, the susceptibility of Atlantic salmon to clinical infections with TSRV has also increased, as noted in 2014 [51]. Infected Atlantic salmon exhibited petechial haemorrhages on their scales, peritoneal surfaces, and visceral fats. There is also evidence of hepatic necrosis with subacute non-suppurative myocarditis and pericarditis, as well as acute posterior uveitis [52]. In 2009, a total of 144 fish (12–13 fish per site) were collected from nine sites in two regions of Tasmania, the Tamar River and Southeast Tasmania, during late spring to early summer. The collected samples were tested via qPCR, which showed a sensitivity and specificity of 95.2% for detecting TSRV, with a prevalence ranging from 6% to 22% in both regions [50].

#### 3.1.4. Pilchard Orthomyxovirus (POMV)

Pilchard orthomyxovirus (POMV) is a single-stranded RNA virus within the *Orthomyxoviridae* family, consisting of eight viral segments that encode ten putative proteins [53]. Although phylogenetic analysis identified infectious salmon anaemia virus (ISAV) as its closest relative, a comparison of six major proteins encoded by the two viruses revealed significant divergence [54]. POMV virus was first identified in 1998 as an incidental finding in wild pilchards (*Sardinops sagax*) collected from waters off the South Australian coast [54]. In 2012, it was subsequently discovered in Atlantic salmon from different fish farms in southern Tasmania [27]. Therefore, there are concerns regarding this virus within the Atlantic salmon industry, and it is classified as a notifiable finfish disease under the regulations of the World Organization for Animal Health (WOAH) [53]. The clinical condition caused by POMV, recently designated salmon orthomyxoviral necrosis (SON), is characterised by symptoms such as lethargy, darkened skin pigmentation, and petechial haemorrhages on ventral regions of the body [55]. The viral load is most prominent in the head kidney and heart, especially during the early stages of infection. However, even if fish recovered from the infection, the virus was still detectable in some tissues and remained at relatively high levels in the gills [56]. Additionally, virus-infected melanomacrophages were found in different tissues, indicating a host immune response against the virus [55]. Diagnostic tools such as reverse transcriptase real-time TaqMan polymerase chain reaction (RT-qPCR) and conventional reverse transcriptase nested PCR (RT-nPCR) were developed to detect POMV in infected fish [54].

#### 3.1.5. Nervous Necrosis Virus (NNV)

Nervous necrosis viruses (NNVs) are classified within the genus *Betanodavirus* and the family *Nodaviridae* [57,58,59,60]. NNV is a small non-enveloped spherical or icosahedral single-stranded RNA virus that can be transmitted either horizontally or vertically [61]. NNVs encode two single-stranded positive-sense RNAs, RNA1 and RNA2, which encode an RNA-dependent RNA polymerase and a structural capsid protein, respectively [61]. NNV has been officially reported in New South Wales, the Northern Territory, Queensland, South Australia, Tasmania, and Western Australia [28]. It is also known to cause viral encephalopathy and retinopathy (VER), which is one of the most significant pathologies of affected fish species [62]. In 1980, VER was first described in larval barramundi (*L. calcarifer*) or Asian sea bass in Australia, and its impact on hatchery production was attributed to a neurological disease [62,63]. Additional signs observed in fish infected with NNV include anorexia, abnormal swimming patterns, fish resting belly-up due to a loss of equilibrium, sporadic protrusion of the head from the water, colour changes such as becoming lighter, blindness, abrasions, over-inflated swim bladders with vacuolation of central nervous tissues, especially the retina, and the presence of crystalline arrays or aggregates of intracytoplasmic inclusions in brain tissues [28,31]. NNV can be detected using validated real-time reverse transcriptase quantitative polymerase chain reaction (RT-qPCR) assays or virus isolation in striped snakehead (SSN-1) cell culture [36].

#### 3.1.6. Infectious Spleen and Kidney Necrosis Virus (ISKNV)

Infectious spleen and kidney necrosis virus (ISKNV) is a species within the *Megalocytivirus* genus, which belongs to the *Iridoviridae* family [64]. *Megalocytivirus* is the most recent addition to the five genera within the *Iridoviridae* family, which consists of large, enveloped, double-stranded DNA viruses [65]. The international trade of live ornamental fish, both freshwater and marine, has been identified as a potential source for the spread of megalocytiviruses, particularly the ISKNV-like virus [66,67]. While megalocytiviruses have not been found in wild fish populations in Australia, several ISKNV-like viruses have been detected in imported ornamental fish and this has caused disease in native species on one occasion [68]. For example, strains such as dwarf gourami *Iridovirus* (DGIV) have been isolated from ornamental fish [67,69,70,71]; notably, DGIV was found in dwarf gourami imported to Australia that later died in commercial aquaria [69]. In 2011, various gourami species imported from multiple countries were found to carry DGIV in both quarantine and post-quarantine facilities [72]. Furthermore, ornamental fish species imported to Australia between 2012 and 2013 showed high mortality rates in quarantine, which was associated with infections with an ISKNV-like virus [73]. Most recently, DGIV was detected at an Australian ornamental fish farm breeding exotic species [72]. The clinical signs of ISKNV infection in these fish included high mortality rates (50–100%), lethargy, poor feeding, respiratory distress, colour changes, exophthalmos, abdominal swelling, and microscopic signs like hypertrophied basophilic cells in haematopoietic tissues, with some affected cells appearing amoeboid [38]. TaqMan qPCR has emerged as a widely used diagnostic tool for screening ISKNV [68].

#### 3.1.7. Bohle Iridovirus (BIV)

Bohle iridovirus (BIV), classified under the genus *Ranavirus* in the family *Iridoviridae*, was initially isolated in Australia from the ornate burrowing frog (*Limnodynastes ornatus*) [74]. Ranavirus is characterised by large, double-stranded DNA virions measuring approximately 150–180 nm in diameter and possessing genomes ranging from 150 to 170 kb [75]. Since its discovery, BIV has been found to infect a variety of hosts, including amphibians, reptiles, and fish such as tilapia (*O. mossambicus*) [76]. The first case of BIV infection in tilapia fry was documented in an aquatic disease research facility in North Queensland, Australia, where it caused 100% mortality [77]. Affected fish displayed erratic, corkscrew-like swimming behaviour, a symptom that led to the disease being referred to as “spinning tilapia” (ST) syndrome [77]. Given Australia’s dependence on robust aquatic populations, the emergence of BIV presents a significant threat to both local aquaculture industries and international trade, especially if the virus spreads to commercially valuable species. At present, no vaccines or targeted treatments exist for BIV, and its diagnosis largely relies on molecular methods, such as PCR, and histopathological examinations [35,78].

#### 3.1.8. Wamena Virus (WV)

Wamena virus (WV), belonging to the family *Iridoviridae*, is generally classified within the genus *Ranavirus* [79]. In Australia, WV was first isolated from a python that had been illegally imported from Irian Jaya [80]. This virus is known to cause severe diseases in fish, amphibians, and snakes, although its distribution appears to be largely confined to the Australasian region [79]. While direct reports of widespread outbreaks within Australia are limited, early detections combined with ecological similarities across regions highlight a credible risk of viral introduction and spread.

### 3.2. Pathogenic Viruses in Prawn

Nine prawn viruses were identified via the search process. Table 2 lists the nomenclature, source, and year of publication for the nine identified viruses.

#### 3.2.1. Infectious Hypodermal and Haematopoietic Necrosis Virus (IHHNV)

Infectious hypodermal and haematopoietic necrosis virus (IHHMV) is currently classified as a potential *Penaeus stylirostris* densovirus (PstDNV), belonging to the genus *Penstylhamaparvovirus* within the subfamily *Hamaparvovirinae* of the *Parvoviridae* family [87,88]. IHHNV is the smallest known penaeid shrimp virus and is characterised by a single-stranded DNA genome approximately 4.0 kb in size with a non-enveloped icosahedral nucleocapsid [89,90]. Two known IHHNV genotypes have been shown to be pathogenic to either Pacific white prawn (*Penaeus* (*Litopenaeus*) *vannamei*) or *P. monodon* [91]. The clinical manifestations in prawns infected with IHHNV include slow floating to the water surface, cannibalism, poor hatching and survival of larvae, opaque abdominal musculature, white to buff lesions on the carapace, runt deformity syndrome, cuticular roughness, cuticular deformities, and mottling of the shell at the abdominal shell plate. Additionally, histologically, virus-infected cells present characteristic intranuclear eosinophilic and haloed Cowdry type A inclusion bodies [91]. Standard diagnostic methods used to detect IHHNV in prawns include histopathological examination, PCR analysis, and ELISA tests [92,93,94].

#### 3.2.2. Gill-Associated Virus (GAV)

Gill-associated viruses (GAVs) are classified as novel members of the family *Roniviridae*, within the genus *Okavirus* [95,96]. GAV belongs to the yellow head virus (YHV) complex and is also referred to as YHV-2 [14]. GAV is characterised by four major structural proteins (~170, 135, 67, and 22 kDa), with the 135 kDa protein being glycosylated; its genome consists of a single-stranded RNA (ssRNA) molecule exceeding 22 kb in length [97,98]. GAV is usually found in eastern Australia and is known to cause chronic infection in both farmed and healthy broodstock *P. monodon* [14]. The virus that emerged in 1996 was less virulent, causing only mid-crop mortality syndrome in farmed *P. monodon* in Australia [99]. The prevalence of GAV infection in healthy *P. monodon* broodstock and farmed prawn in Australia has been reported to be close to 100%. While viruses can be transmitted through injection or exposure to moribund prawn via oral routes, outbreaks in ponds are most likely driven by the amplification of viral loads due to environmental stress [100]. The other four known viral genotypes were exclusively detected in healthy *P. monodon* in Asia, where they are prevalent in various regions but not associated with disease [101]. The pathological signs observed due to this virus include reddening of the body and appendages, pink to yellow coloration of the gills, and the necrosis of lymphoid organs [102]. According to Nobel et al. (2020), GAV infection is affected by sex, since female prawns have a higher prevalence of infection (50.2%) compared to their male counterparts (41.8%). Thus, one of the factors affecting the selection of females for breeding is that GAV can be transferred vertically and presented on egg surfaces. In addition, selecting host families with lower viral infection loads will likely lead to lower GAV prevalence [103]. The diagnostic methods for detecting GAV in clinical samples include in situ hybridisation (ISH), RT-nested PCR, and RT-qPCR [104,105,106,107].

#### 3.2.3. Macrobrachium Rosenbergii Nodavirus (MrNV)

Macrobrachium rosenbergii nodavirus (MrNV), a member of the *Nodaviridae* family [108], is a non-enveloped, icosahedral virus (27 nm in diameter) with a genome consisting of two positive-sense single-stranded RNA fragments: RNA 1 and RNA 2 [109]. It causes white muscle disease/white tail disease (WTD) in giant freshwater prawn (*Macrobrachium rosenbergii*) [108] and was first reported on Guadeloupe Island in 1997 [110] and later in Australia [111]. It is known to be a limiting factor in crustacean species development, especially in *M. rosenbergii* larvae and juveniles, where it had a 100% mortality rate. *Mr*NV targets the haemocytes and myonuclei of the prawn’s lower abdomen before spreading. Infection occurs when a virus binds to a host cell receptor through the caveolin-mediated endocytosis pathway. This virus is spread through the haemolymph circulatory system and is found in almost every organ except the eyestalks and hepatopancreas [112]. Several diagnostic techniques have been developed for detecting MrNV, including a sandwich enzyme-linked immunosorbent assay (S-ELISA), dot blot hybridisation, in situ hybridisation, and RT-PCR [113].

#### 3.2.4. White Spot Syndrome Virus (WSSV)

White spot syndrome virus (WSSV) has been assigned to the newly established genus *Whispovirus* within the family *Nimaviridae* on the basis of its distinctive morphology and genomic composition [114,115,116]. WSSV is a large, enveloped, double-stranded DNA virus that is known to cause panzootic white spot disease (WSD), which affects prawn aquaculture [117]. The first reported cases of WSD date back to 1992 in mainland China and Taiwan, and it had spread throughout Asia by the following decade [82]. Australia remained free from WSD until 2016, when there was a WSSV-associated disease outbreak in a prawn farm near Brisbane, southeastern Queensland. The clinical signs of the disease included reddish pink and white calcified spots on the prawns [82]. According to the Department of Agriculture (2019), prawns infected with WSSV showed white spots embedded within the exoskeleton, a loosened carapace, and anorexia, since the GIT was empty, along with the delayed clotting of haemolymph and excessive fouling of the gills [118]. WSD has caused large economic losses due to the high mortality of penaeids through the ingestion of infected tissues or direct contact with infected individuals. Furthermore, WSSV is likely transmitted vertically through infected females to their offspring [82]. For the diagnosis of WSSV, PCR has been shown to be a highly accurate method, even in asymptomatic prawns [119].

#### 3.2.5. Whenzhou (Syn. Wenzhou) Shrimp Virus-2 (When-2)

Whenzhou (syn. wenzhou) shrimp virus-2 (When-2) was first officially identified in an unclassified segmented virus 3 RNA sequence of *P. monodon* in Wenzhou, Zheijiang Province, China [120]. Most recently, an uncharacterised When-2 viral genome was also identified in *P. monodon* stocks in Australia [121]. A study reported the total prevalence of When-2 to be 2.9% (220/7472), with the majority of positive detections occurring on the East Coast of Queensland (199 out of 220 samples), indicating its potential presence in Australian stocks [20]. However, the impact of this virus on the health and productivity of *P. monodon* has yet to be thoroughly investigated [20]. Additionally, the detection of *Hepandenovirus* in the replicative phase of GAV suggests that similar techniques could be employed to identify viruses with DNA genomes [121]. Notably, quantitative PCR is the most widely used technique for the detection of When-2 [20].

#### 3.2.6. Monodon Baculovirus (MBV)

Monodon baculovirus (MBV), also referred to as nuclear polyhedrosis virus (NPV), is classified within the genus *Nucleopolyhedrovirus* of the *Baculoviridae* family [122]. NPV has a double-stranded circular DNA genome of 80–160 kb pairs and a rod-shaped, enveloped particle that is often occluded within proteinaceous bodies [123]. In Australia, NPV has been detected in both cultured *P. monodon* and wild *P. merguiensis* [124], but it is not thought to be pathogenic, as it is usually contracted in hatcheries where prawns are cultured under sub-optimal conditions. The primary target organs of NPV are the hepatopancreas and anterior midgut [125]. Prawns affected by NPV are lethargic, anorexic, and foul smelling; dysfunction in the hepatopancreas, anterior midgut, and other organs is also observed. In addition, co-infection with bacteria can also occur, resulting in hypertrophied nuclei containing spherical occlusion bodies [123,126]. Good management practices, such as washing fertilised eggs and nauplii with clean seawater, have been recommended to control MBV or avoid its spread within the cultured stock [127]. Furthermore, rapid molecular detection methods, including PCR and DNA hybridisation-based genomic probes (either in situ or dot blot), have been developed for the early identification of MBV in prawn samples [128,129,130].

#### 3.2.7. Mourilyan Virus (MoV)

Mourilyan virus (MoV) is classified within the family *Phenuiviridae*, genus *Wenrivirus*, and order *Bunyavirale* [131]. MoV is an enveloped negative-sense ssRNA bunyavirus that is 85–100 nm in diameter and contains four segmented genomes [132]. The virus is known for infecting *P. monodon* and *P. japonicus*, which are both farmed in eastern Australia [133,134]. Prawns infected with MoV show damage to mesodermal and ectodermal tissues, accompanied by the formation of enveloped, ovoid-shaped particles [84]. In *P. japonicus* reared in tanks after grow-out in farm ponds, increased MoV infection loads are correlated with higher mortality rates [135]. Additionally, recent experimental challenge studies have validated the pathogenicity of the virus [84]. The detection of MoV in prawn samples is primarily conducted using the TaqMan qRT-PCR assay [136].

#### 3.2.8. Hepatopancreatic Parvovirus (HPV)

Hepatopancreatic parvovirus (HPV) is classified within the *Parvoviridae* family based on its virion characteristics [137]. HPV is a non-enveloped, icosahedral virus with an average diameter ranging from 22 to 24 nm. The HPV virus genome is a single linear DNA strand approximately 6 kb in size [138]. HPV was first reported in 1984 in wild *P. merguiensis* and *P. indicus* from Singapore [139]. In Australia, the earliest detection occurred in 1985, when HPV was identified in *Penaeus esculentus* from Moreton Bay and the Gulf of Carpentaria [140]. Subsequently, HPV was found in *P. merguiensis* in 1989 and later in *P. monodon* and *P. japonicus* [141,142,143]. This virus is associated with severe outbreaks, where it causes mortality rates of up to 50–100% as well as stunting [142,144,145]. Furthermore, HPV infection has been linked to reduced growth rates in juvenile prawns [144]. qPCR has been demonstrated to be a valuable tool for accurately assessing the HPV levels in broodstock [146].

#### 3.2.9. Yellow Head Virus Genotype 7 (YHV7)

Yellow head virus genotype 7 (YHV7) is a recently identified strain within the YHV complex. YHV7 was first detected in *P. monodon* from the Joseph Bonaparte Gulf (JBG) in northern Australia [86]. Phylogenetic analysis of the open reading frame 1b (ORF1b) gene revealed that YHV-7 is the seventh distinct genotype in the YHV complex and has the highest genetic similarity to YHV-1 [86]. The pathogenicity of YHV-7 was confirmed through injection, co-habitation, and feeding in *P. monodon*, which resulted in approximately 60% cumulative mortality at 28 days post-infection [147]. Additionally, YHV-7 has also been associated with sporadic disease outbreaks in pond-reared *P. monodon* [148]. To detect YHV-7 in prawn populations, sensitive and specific diagnostic methods, including TaqMan qRT-PCR and conventional nested PCR assays targeting the ORF1b gene, have been developed [148].

### 3.3. Comparative Insights on Viruses in Fish and Prawn

A comparative assessment of the viruses affecting Australian aquaculture reveals distinct patterns in the emergence, endemicity, and trade risk. Among finfish pathogens, EHNV, TSRV, and BIV are long-established and endemic, causing localised but significant losses [23,52,77]. In contrast, NNV and POMV are increasingly widespread and linked to severe disease outbreaks, particularly in hatchery systems. CyHV-2, though introduced via the ornamental trade, exemplifies the risks posed by imported live animals. ISKNV and WV, while not yet established, represent emerging threats with high incursion potential.

In prawn aquaculture, WSSV is the most economically disruptive pathogen, with its 2016 outbreak causing over AUD 23 million in damages [149]. Endemic viruses such as IHHNV and GAV remain prevalent in wild-sourced broodstock, complicating control efforts [150,151]. YHV-7, MoV, and MrNV are emerging concerns due to their virulence and limited diagnostic capacity [136,148,152]. Other detected viruses, including When-2, MBV, and HPV, vary in impact but highlight the growing complexity of disease surveillance.

### 3.4. Impacts of Viral Diseases

In the next section, we will explore the economic impacts of viral diseases on Australian fish and prawn aquaculture, with an emphasis on the direct and indirect costs, trade implications, and mitigation strategies. A summary comparing the economic impact of major viruses is presented Table 3.

#### 3.4.1. Direct Economic Losses

Viral diseases such as TSRV, KHV, and WSSV have had devastating impacts on Australian aquaculture production [26,162]. A notable outbreak occurred in December 2016, when WSSV spread in commercial *P. monodon* prawn farms along the Logan River in Queensland, northeast Australia. This led to the complete loss of prawn production in the region, either directly due to the virus or indirectly through quarantine measures that resulted in the destruction of affected farms [153]. This outbreak had a significant and immediate financial impact on prawn farming in the region, directly affecting five farming families and resulting in the destruction of stock across grow-out ponds, hatcheries, and breeding programmes, with losses estimated at AUD 23.5 million for the 2016–2017 period [154]. Additionally, two farms that managed breeding programmes for *P. monodon* in their private hatcheries experienced a complete loss of spawning stocks, severely disrupting their genetic development capabilities [154], while Gold Coast Marine Aquaculture, one of the Australia’s largest prawn farming enterprises, reported a loss of 25 million prawns due to mandatory destocking under national eradication efforts [155]. The first farm to test positive for WSSV reported stock losses exceeding AUD 1 million, despite receiving an AUD 400,000 government support package to mitigate the financial impact [156].

Beyond prawns, the broader aquaculture industry also faces challenges, as Barramundi farming, which initially thrived as an alternative, was undermined by nodavirus outbreaks that caused high larval and juvenile mortality, significant financial losses, and the insolvency of the country’s first Barramundi hatchery [162,163]. The direct financial losses primarily stem from mass mortalities among farmed aquatic species, which not only reduce overall yields but also disrupt farming cycles.

#### 3.4.2. Indirect Economic Costs

The indirect economic costs resulting from viral infections in fish and prawn aquaculture in Australia extend beyond the immediate loss of stock. Infected fish and prawns often exhibit poor feed conversion ratios, requiring farmers to spend more on feed for diminished output. Farmers also face significant financial strain from the delays in production cycles caused by the need to restock and rebuild populations, which can take months or even years, leading to lost revenue and disrupted supply chains [157,158]. This inefficiency translates into higher production costs and lower profitability [159]. Restrictions on the movement and trade of aquatic products imposed during outbreaks further exacerbate the economic losses by limiting market access and increasing compliance costs [164]. Additionally, disease outbreaks can cause significant disruptions to production cycles. Farmers may need to halt operations to manage and eradicate diseases, leading to delays in restocking and rebuilding populations [165]. For example, the emergence of white spot syndrome in Queensland’s prawn farms in 2016 led to an immediate cessation of operations to treat the disease. Eradication efforts included culling infected stock and implementing strict biosecurity measures, resulting in delays in restocking and prolonged production downtime [149].

#### 3.4.3. Impacts on Trade and Market Access

Viral diseases also disrupt international trade by triggering trade restrictions and a loss of consumer confidence. Countries importing Australian aquaculture products may impose stringent sanitary requirements or outright bans on exports from regions experiencing outbreaks. For instance, the presence of WSSV in Australian prawns has previously led to temporary trade bans, significantly affecting export revenues and damaging Australia’s reputation as a reliable supplier [153,160]. Beyond direct trade restrictions, viral outbreaks also create market uncertainty. Consumers, wary of potential health risks, may shift their preferences away from products originating from affected regions, even after the outbreak is controlled. Exporters may also face increased costs related to compliance with new sanitary regulations, including certifications, testing, and biosecurity audits, which can be resource-intensive and time-consuming processes [166]. Outbreaks can reduce the availability of high-quality products, leading to price volatility and reduced competitiveness against imported alternatives. This dynamic can erode market share and profitability for Australian aquaculture producers in the long term.

#### 3.4.4. Socioeconomic Implications

Viral diseases have profound socioeconomic implications for Australia’s aquaculture industry, particularly fish and prawn farming. The white spot disease outbreak in Queensland’s Logan River region in 2016 resulted in an estimated farm gate loss of approximately AUD 24 million, representing around 25% of the country’s aquaculture prawn sector at that time [161]. Such outbreaks can be associated with high mortality rates, with WSSV causing up to 100% mortality in infected prawns within 3–10 days, severely impacting production [167]. These losses place a significant financial strain on farmers, many of whom struggle with income loss and debt due to production failures [154]. Additionally, disease outbreaks disrupt trade and markets; for example, following the detection of WSSV in 2016, movement restrictions and heightened biosecurity measures were imposed, affecting supply chains and international exports [149]. In response, the Australian government implemented strict movement restrictions and decontamination procedures to control outbreaks, aiming to mitigate the long-term industry damage [168].

### 3.5. Biosecurity Policies and Solutions for Managing Viral Diseases

Viral diseases are a significant threat to Australian fish and prawn aquaculture, causing severe economic losses and threatening sector sustainability. The implementation of robust biosecurity measures is essential for the future growth of aquaculture, as ensuring the health of aquatic species is essential for meeting the global demand for food [169]. Australia’s biosecurity focuses on animal health, public health, and the integration of best management practices at all the production stages—from broodstock facilities to exports [170]. National strategies such as AQUAPLAN and AQUAVETPLAN have been central to this effort [171]. These strategies were first implemented in 1987 to address aquatic animal diseases [172] and later evolved through multiple revisions [173,174,175]. AQUAPLAN, developed by the Department of Agriculture, Fisheries and Forestry, outlines national priorities for aquatic animal health, while AQUAVETPLAN provides technical response guidelines during disease outbreaks [176,177]. Moreover, Animal Health Australia (AHA) plays a key role in the emergency response coordination and cost-sharing arrangements between the industry and government [178].

#### 3.5.1. Mitigation Strategies

Investments in disease prevention, early detection, and effective management are crucial to minimising the economic impacts of viral diseases. The development of robust biosecurity protocols, vaccination programmes, and genetic improvements for disease-resistant strains has shown promise in reducing the losses in aquaculture globally [179], but these measures are not well documented in Australia. Advancements in molecular diagnostics and next-generation sequencing have enhanced our ability to detect and monitor viral pathogens in aquaculture systems [14,180]. Ongoing investment in research, particularly in the development of RNAi-based therapies for viral diseases, may have an impact on the long-term sustainability of aquaculture [181]. Early detection allows for swift intervention, minimising the spread of disease and reducing the overall economic impact. Additionally, international collaborations and knowledge-sharing initiatives provide Australian aquaculture stakeholders with access to cutting-edge research and best practices in disease management [182]. Moreover, implementing comprehensive insurance schemes and financial support mechanisms for farmers affected by outbreaks can also help to mitigate economic losses. AHA has been working with industry sectors and the government to determine the emergency response and cost-sharing arrangements for future aquatic emergencies [183]. However, although AHA’s efforts to establish emergency response frameworks are crucial, implementing equitable cost-sharing and compensation remains a challenge.

#### 3.5.2. Quarantine and Import Controls

Quarantine and import controls are the first line of defence against the introduction of viral pathogens into Australian aquaculture. The Biosecurity Act 2015 provides a robust framework for screening aquatic species and related products [184,185]. Diagnostic tests, such as PCR, are conducted during this period for high-sensitivity pathogen detection [14]. Australia also restricts the importation of certain feed ingredients and other biological materials to minimise the contamination risks. For example, regulations on frozen prawn imports are crucial to mitigating threat of WSSV, which devastated several farms in Queensland during a 2016 outbreak [186]. The Federal Government almost entirely relied on an at-border testing programme for risk mitigation in November/December 2016 [187]. However, this reliance on border testing revealed vulnerabilities, prompting updated risk analyses and surveillance protocols.

#### 3.5.3. Surveillance and Early Detection

DAFF emphasises that surveillance is essential for early disease detection, demonstrating Australia’s disease status, and maintaining market access [188]. Advancements in diagnostic technologies have further enhanced early detection capabilities [189]. Australian biotech company Genics developed Shrimp MultiPath2.0, a technology capable of detecting 18 shrimp diseases and identifying genetic variations in a single test. Additionally, the Commonwealth Scientific and Industrial Research Organization (CSIRO) validated PCR tests for detecting WSSV, confirming their high accuracy in samples from apparently healthy prawns [119]. On the other hand, a non-invasive, environmental DNA (eDNA) analysis technique has become a valuable tool for detecting viral pathogens in water samples [190] and allows for continuous monitoring without disturbing aquaculture stocks. While the technical capabilities are effective, ensuring consistent adoption across farms and maintaining long-term funding remain hurdles.

#### 3.5.4. Farm-Level Biosecurity Measures

Farm-level biosecurity practices are critical to minimising the risk of viral transmission within and between aquaculture facilities. Hatcheries often implement water filtration systems and use treated water to reduce the contamination risks [191]. Regular cleaning and disinfection of tanks, nets, and feeding equipment can eliminate viral particles and prevent their spread. To reduce the risk of introducing viruses into aquatic environments, farms should obtain disease-free stock and feed that is free from pathogens [168]. Training and raising awareness among farm staff about biosecurity practices ensure consistent implementation and adherence. These practices, when consistently implemented, significantly lower the risk of viral outbreaks and contribute to farm sustainability [179,192].

#### 3.5.5. Emergency Response and Contingency Planning

Australia’s biosecurity framework includes various emergency response plans to address viral disease outbreaks [192]. In the event of an outbreak, affected farms are immediately isolated to prevent contact with surrounding facilities. The culling of infected stock is another standard measure during severe outbreaks, followed by the safe disposal of carcasses. The farms are disinfected, and all the equipment is sanitised to eliminate residual pathogens. Temporary bans on the movement of live aquatic animals and related materials have also been enforced in affected areas [193]. During the 2016 WSSV outbreak in Queensland, rapid response teams worked with local farmers to limit the virus’s spread, demonstrating the importance of coordinated action [153,185]. However, contingency planning must also address post-outbreak recovery support for affected farmers.

#### 3.5.6. Research and Innovation

The Cooperative Research Centre for Developing Northern Australia (CRCNA) has conducted studies to understand the impact of pathogens on prawn health and survival, with a particular focus on environmental factors, including water quality and temperature [194]. Additionally, James Cook University is advancing the use of eDNA sampling methods, which serve as early detection tools for pathogens on prawn farms, potentially reducing biosecurity costs and preventing outbreaks [190]. Furthermore, treatment with chemicals, like trichlorfon, has been validated for controlling white spot disease, enhancing biosecurity protocols on Australian prawn farms and contributing to effective management and prevention strategies that are used to safeguard the Australian aquaculture industry [195]. Additionally, the CSIRO in Australia developed Shrimp MultiPath technology, which helps to detect 13 commercially significant prawn diseases, including IHHNV and WSSV [196]. However, broader industry uptake of these solutions is needed to ensure system-wide resilience.

#### 3.5.7. Building Resilience Through Education and Training

To enhance the national biosecurity capacity, the Australian government, in collaboration with Charles Sturt University, established the Biosecurity Training Centre in 2022, offering professional training and specialised biosecurity programmes aimed at building sector-wide expertise [197]. Complementing this initiative, the National Biosecurity Training Hub provides an accessible online platform with a wide range of aquatic biosecurity training resources, although maintaining high levels of participation across remote farming communities remains a challenge [198]. To further strengthen biosecurity awareness, the CRCNA developed a biosecurity training video specifically for aquaculture, aimed at improving on-farm biosecurity practices in northern Australia [199]. Additionally, the Australian government provides guidelines for creating farm-specific biosecurity plans, emphasising staff training to manage disease prevention and outbreaks [192]. To further support industry needs, the FRDC offers an Aquatic Animal Health and Biosecurity Training Scheme to support the industry’s health management training needs [200]. Together, these initiatives represent a comprehensive approach, though ongoing challenges related to engagement, consistency, and regional implementation highlight the need for continuous improvement.

### 3.6. Knowledge Gaps and Recommendations

Viral diseases pose a major threat to the economic viability and environmental sustainability of the Australian fish and prawn industries, as they can lead to stock losses, increased operational costs, export restrictions, and reduced industry confidence. Efforts to address these challenges are hindered by key knowledge gaps, including the lack of early diagnostic tools, insufficient understanding of viral transmission dynamics, and inadequate assessment of economic impacts.

To safeguard the industry’s sustainability, priority should be given to developing rapid, accessible molecular diagnostics and establishing a coordinated national surveillance framework. Strengthening biosecurity through advanced water treatment systems and selective breeding for genetic resistance is equally critical. Targeted research into the effects of environmental changes, particularly temperature fluctuations, on viral emergence and transmission is urgently needed. Understanding the temperature-dependent viral dynamics is vital for refining predictive models and adapting disease mitigation strategies under climate change pressures.

Finally, integrating stakeholder engagement, economic resilience planning, and support for small-scale producers into biosecurity responses will be key to strengthening industry-wide collaboration. Through these actions, Australia can effectively manage the economic and ecological consequences of viral threats, ensuring the long-term sustainability of its aquaculture sectors.

## Figures and Tables

**Table 1:** Pathogenic viruses reported in the literature from fish species found in Australia.

Pathogenic Virus	Viral Genus	Viral Family	Type	Susceptible Fish Species	Origin	Year of Study	Impact Level	References
EHNV	*Ranavirus*	*Iridoviridae*	dsDNA	Wild redfin (*Perca fluviatilis*)	Victoria, Australia	July 2007 and June 2011	High	[23]
CyHV2	*Cyvirus*	*Alloherpesviridae*	dsDNA	Goldfish (*Cyprinus carpio*)	Western Australia	*	Moderate	[24]
TSRV	*Aquareovirus*	*Reoviridae*	dsRNA	farmed Atlantic salmon (*Salmo salar*)	Tasmania	Since 2005	Moderate	[25,26]
POMV	Unclassified	*Orthomyxoviridae*	ssRNA	Atlantic salmon	Southern Tasmania	2012	High	[27]
NNV	*Betanodavirus*	*Nodaviridae*	ssRNA	Barramundi (*Lates calcarifer*)	New South Wales, Northern Territory, Queensland, South Australia, Tasmania, and Western Australia	*	High	[28]
ISKNV	*Megalocytivirus*	*Iridoviridae*	dsDNA	Ornamental fish	Exotic—not recorded in Australia	2008 and 2011	Potential threat	[29]
BIV	*Ranavirus*	*Iridoviridae*	dsDNA	Tilapia fry (*Oreochromis mossambicus*)	North Queensland, Australia	*	Moderate	[30]

* Year the study was performed was not mentioned. Table 1 is a summary of the major viral pathogens affecting fish species in Australia. The table highlights the pathogen type, host range, and geographic distribution, with a classification of the impact level based on the known or potential disease severity and economic consequences.

**Table 2:** Pathogenic viruses reported in the literature from prawn species found in Australia.

Pathogenic Virus	Viral Genus	Viral Family	Genome Type	Susceptible Prawn Species	Origin	Year of Study	Impact Level	References
IHHNV	*Penstylhamaparvovirus*	*Parvoviridae*	ssDNA	*P. monodon*	Northern Territory and Queensland	July 2018 and October 2020	High	[14,20]
GAV	*Okavirus*	*Roniviridae*	ssRNA	*P. monodon*	Queensland	July 2018 and October 2020	Moderate	[14,20]
MrNV	Unclassified	*Nodaviridae*	ssRNA	*Giant freshwater prawn* *Macrobrachium rosenbergii*	Queensland	During January and March 2020	Moderate	[81]
WSSV	*Whispovrius*	*Nimaviridae*	dsDNA	Penaeid shrimps	South-Eastern Queensland	2017	Very high	[82]
When-2	Unclassified	Unclassified	ssRNA	*P. monodon*	Wenzhou in Zhejiang province, China	Between July 2018 and October 2020	Unknown	[20]
MVB	*Nucleopolyhedrovirus*	*Baculoviridae*	dsDNA	*P. monodon*	Australia	*	Unknown	[83]
MoV	*Wenrivirus*	*Phenuiviridae*	ssRNA	*P. monodon* and Kuruma shrimp (*Penaeus japonicus*)	Eastern Australia	*	Moderate	[84]
HPV	*Densoviruses*	*Parvoviridae*	ssDNA	*P. monodon*, *P. japonicus*, Brown tiger prawn (*P. esculentus*), Indian white prawn (*P. indicus*), *P. merguiensis*	Moreton Bay Gulf of Carpentaria	2005	Moderate	[85]
YHV-7	*Unclassified*	*Unclassified*	-	*P. monodon*	Joseph Bonaparte Gulf in northern Australia	November 2012	High	[86]

* Year the study was performed was not mentioned. Table 2 is an overview of the key viral pathogens affecting prawn species in Australian aquaculture, including the genome type, host specificity, and geographical distribution. Pathogens such as WSSV and IHHNV are marked as high impact due to their widespread occurrence and severe economic implications.

**Table 3:** Comparative summary of the economic impact of major viral diseases in fish and prawn aquaculture in Australia.

Virus	Year of Major Outbreak	Affected Species	Direct Economic Losses	Indirect Economic Costs	Trade and Market Impact	Socioeconomic Implications	References
WSSV	2016	*P. monodon*	Estimated AUD 23.5 million loss (2016–2017); loss of 25 million prawns; single farm loss > AUD 1 million despite AUD 400 k support	Restocking delays, poor feed conversion, downtime, higher production cost	Export bans, market uncertainty, compliance costs, price volatility	AUD 24 million farm gate loss (~25% of sector); 100% mortality within 3–10 days; income loss and debt in farmers	[147,149,153,154,155,156,157,158,159,160,161]
NNV	Recurrent (no fixed date)	Barramundi (larvae and juveniles)	Insolvency of first Barramundi hatchery in Australia	High larval mortality; delays in production cycles	Market instability due to supply chain disruption	Disruption of breeding; financial loss for hatcheries	[162,163]
TSRV	Endemic (concerns resurfacing recently)	*S. salar*	Not quantified; concerns emerging from association with diseased fish	Potential productivity loss under stress conditions; diagnostic and surveillance costs	No documented trade bans, but growing industry concern	Industry-driven monitoring and management; vertical and horizontal transmission risks	[26]

## Data Availability

No external link is applicable for the data.

## Supplementary Materials

The following supporting information can be downloaded at https://www.mdpi.com/article/10.3390/v17050692/s1, Table S1: PRISMA checklist.
